# Therapeutic potential and translational challenges of extracellular vesicles in neonatal medicine

**DOI:** 10.1002/btm2.70093

**Published:** 2025-11-25

**Authors:** Ali M. Atoom, Media Hamed‐Ahmed, Shaker Al‐Hasnaawei, H. Malathi, Laxmidhar Maharana, Anima Nanda, Vimal Arora, Ashish Singh‐Chauhan, Elham Poursoltani

**Affiliations:** ^1^ Faculty of Allied Medical Sciences, Hourani Center for Applied Scientific Research Al‐Ahliyya Amman University Amman Jordan; ^2^ Department of Anesthesia Techniques, Health and Medical Techniques College Alnoor University Mosul Iraq; ^3^ College of Pharmacy The Islamic University Najaf Iraq; ^4^ Department of Medical Analysis, Medical Laboratory Technique College The Islamic University of Al Diwaniyah Al Diwaniyah Iraq; ^5^ Department of Biotechnology and Genetics, School of Sciences JAIN (Deemed to be University) Bangalore Karnataka India; ^6^ Department of Pharmaceutical Sciences Siksha ‘O’ Anusandhan (Deemed to be University) Bhubaneswar Odisha India; ^7^ Department of Biomedical Sathyabama Institute of Science and Technology Chennai Tamil Nadu India; ^8^ University Institute of Pharma Sciences, Chandigarh University Mohali Punjab India; ^9^ Uttaranchal Institute of Pharmaceutical Sciences, Division of Research and Innovation Uttaranchal University Dehradun Uttarakhand India; ^10^ Clinical Research Development Unit Bohlool Hospital, Gonabad University of Medical Sciences Gonabad Iran

**Keywords:** clinical translation, extracellular vesicles, mesenchymal stem cells, neonatal therapy, regenerative medicine

## Abstract

Extracellular vesicles (EVs) have emerged as promising therapeutic candidates for a range of neonatal diseases, including sepsis, necrotizing enterocolitis, hypoxic–ischemic encephalopathy (HIE), and bronchopulmonary dysplasia (BPD). Derived from diverse sources such as mesenchymal stem cells, breast milk, and even non‐animal systems, EVs exhibit potent anti‐inflammatory, immunomodulatory, and tissue‐regenerative properties. Preclinical studies in neonatal models demonstrate their ability to reduce inflammation, preserve epithelial and endothelial barrier integrity, modulate immune cell phenotypes, and mitigate organ damage. Despite these encouraging findings, several critical barriers hinder their clinical translation. Challenges include incomplete characterization of EV molecular cargo, variability in isolation and quantification methods, lack of standardized dosing protocols, and limited safety data, particularly regarding procoagulant activity and thrombotic risk. The development of standardized, reproducible isolation techniques, rigorous molecular profiling, and GLP‐compliant safety assessments is essential to establish clinical readiness. Current early‐phase clinical trials targeting neonatal BPD, prevention of prematurity‐related brain injury, and HIE indicate growing translational momentum. If these challenges are addressed, EV‐based therapeutics could transform neonatal care, reducing mortality and long‐term disability in vulnerable preterm and term infants.

## Graphical abstract

Potential therapeutic effects of extracellular vesicles (EVs) on major neonatal diseases. EVs exert anti‐inflammatory, anti‐apoptotic, and regenerative actions in neonatal sepsis, necrotizing enterocolitis, hypoxic–ischemic encephalopathy, and respiratory distress syndrome. Mechanistic pathways involve modulation of cytokines, immune cell activation, neuroprotection, angiogenesis, and barrier integrity.

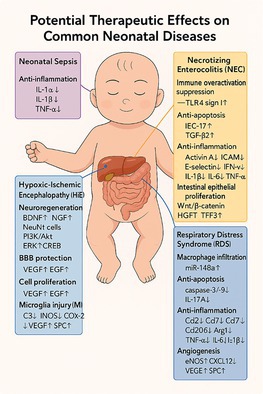

**Abbreviations**
AEC‐II: type II alveolar epithelial cell
AI: artificial intelligence
APP: amyloid precursor protein
ATP: adenosine triphosphate
BBB: blood–brain barrier
BDNF: brain‐derived neurotrophic factor
BPD: bronchopulmonary dysplasia
CD: cluster of differentiation
circRNA: circular RNA
CKD: chronic kidney disease
CRNDE: colorectal neoplasia differentially expressed (lncRNA)
CSF: cerebrospinal fluid
DANCR: differentiation antagonizing non‐protein coding RNA
DIC: disseminated intravascular coagulation
DNA: deoxyribonucleic acid
DOX: doxorubicin
EGF: epidermal growth factor
EV: extracellular vesicle
EVs: extracellular vesicles
EV‐YF1: Y‐RNA fragment 1 in exosomes
GDNF: glial cell line‐derived neurotrophic factor
GAS5: growth arrest–specific 5 (lncRNA)
GPC1: glypican‐1
GPX: glutathione peroxidase
H&E: hematoxylin and eosin
HIF‐1α: hypoxia‐inducible factor 1‐alpha
HIE: hypoxic–ischemic encephalopathy
hAEC‐EVs: human amniotic epithelial cell–derived extracellular vesicles
HSP: heat shock protein
HSP70: heat shock protein 70
HSP72: heat shock protein 72
HSF: heat shock factor
IKK: IκB kinase
IL: interleukin
IL‐1RA: interleukin‐1 receptor antagonist
ISEV: International Society for Extracellular Vesicles
IGF‐1: insulin‐like growth factor 1
lncRNA: long non‐coding RNA
MALAT1: metastasis‐associated lung adenocarcinoma transcript 1
MAPK: mitogen‐activated protein kinase
MDA: malondialdehyde
MET: mesenchymal–epithelial transition factor
miRNA: microRNA
MISEV: minimal information for studies of extracellular vesicles
miR: microRNA (specific number indicates isoform, e.g., miR‐146a)
mRNA: messenger RNA
MSC: mesenchymal stem cell
MSC‐EVs: mesenchymal stem cell‐derived extracellular vesicles
MRP: multidrug resistance‐associated protein
mTOR: mammalian target of rapamycin
NEC: necrotizing enterocolitis
NGF: nerve growth factor
NF‐κB: nuclear factor kappa B
NLRP3: NOD‐like receptor family pyrin domain‐containing 3
OMV: outer membrane vesicle
piRNA: PIWI‐interacting RNA
PGC‐1α: peroxisome proliferator‐activated receptor gamma coactivator 1‐alpha
PPARγ: peroxisome proliferator‐activated receptor gamma
PTEN: phosphatase and tensin homolog
RDS: respiratory distress syndrome
RNA: ribonucleic acid
ROS: reactive oxygen species
SIRT3: sirtuin 3
SOD: superoxide dismutase
SRA1: steroid receptor RNA activator 1
STAT3: signal transducer and activator of transcription 3
siRNA: small interfering RNA
snRNA: small nuclear RNA
snoRNA: small nucleolar RNA
TFAM: mitochondrial transcription factor A
TGF‐*β*
: transforming growth factor‐beta
Th: T‐helper cell
TNF: tumor necrosis factor
TNF‐*α*
: tumor necrosis factor‐alpha
TPG: therapeutic product guideline
TRAF: TNF receptor‐associated factor
Treg: regulatory T cell
TSG‐6: tumor necrosis factor‐stimulated gene 6 protein
VEGF: vascular endothelial growth factor
WNT: wingless/Integrated (signaling pathway)
Y‐RNA: small noncoding RNA of Ro RNP family

**Translational Impact Statement**

Extracellular vesicles (EVs) represent a breakthrough in neonatal therapy, offering a safe, cell‐free alternative to stem cell treatments. Their ability to reduce inflammation, protect tissues, and support repair positions them as powerful tools for conditions such as sepsis, NEC, HIE, and BPD. However, clinical success depends on standardizing EV isolation, characterization, and dosing while ensuring safety and reproducibility. Overcoming these challenges could enable EV‐based therapies to become transformative treatments that reduce mortality and long‐term complications in premature and critically ill infants.

## 1 INTRODUCTION

Pathological conditions in newborns, particularly those born prematurely, are often linked to the physiological immaturity associated with early delivery and its subsequent complications. Preterm birth significantly predisposes infants to acute neonatal illnesses and elevates the likelihood of unfavorable health outcomes due to the onset of severe disorders. According to data from the World Health Organization, the global incidence of preterm delivery ranges between approximately 4% and 15% of all live births, with notable variations between countries.^1^

Neonatal pathologies encompass a broad spectrum of disorders that manifest within the first 28 days of life and, depending on their severity, can result in lasting functional impairment or profound disability. These conditions typically involve multiple organ systems and are frequently accompanied by systemic inflammatory responses. Among the most prevalent are hypoxic–ischemic encephalopathy (HIE), respiratory distress syndrome (RDS), bronchopulmonary dysplasia (BPD), neonatal sepsis, and necrotizing enterocolitis (NEC).^2^

Although advances in neonatal intensive care have improved survival rates, long‐term morbidities, such as chronic lung disease, neurological injury, and persistent pulmonary hypertension, remain widespread. These complications may lead to irreversible damage affecting the nervous system, pulmonary function, cardiovascular health, and metabolic regulation.^3^, ^4^ The management of such complex conditions requires an integrated, multidisciplinary approach that brings together expertise from neonatology, anesthesiology, pediatrics, pediatric surgery, neuropathology, and rehabilitation medicine. This collaborative strategy facilitates early and accurate diagnosis, the initiation of evidence‐based interventions, and the incorporation of novel therapeutic approaches alongside conventional treatments.^3^

In the early perinatal period, pharmacological therapy remains a cornerstone of clinical management. However, this approach faces significant challenges, including the limited number of medications specifically designed for neonatal physiology, the potential for adverse side effects, and inconsistent therapeutic efficacy.^5^ Due to the scarcity of formulations validated for newborns, clinicians often rely on adapting adult medications, which have not been adequately tested for safety, dosing accuracy, or pharmacological suitability in neonates. This practice increases the risk of inappropriate drug administration, dosing errors, suboptimal timing, and potentially severe complications that may have lifelong consequences. Moreover, the physiological immaturity of preterm infants complicates the assessment of drug pharmacokinetics and pharmacodynamics, as these parameters differ considerably from those in older children and adults.^6^

Current evidence highlights a substantial gap in the systematic evaluation of neonatal pharmacotherapy, particularly in relation to antibiotics and other high‐risk medications. This gap underscores the urgent need for rigorous categorization, validation, and optimization of pharmacologic regimens for newborn patients.^7^

Given these limitations, there is growing interest in the development of innovative treatment modalities for acute neonatal diseases aimed at improving clinical outcomes and minimizing long‐term disability.^8^ Preclinical and clinical investigations have demonstrated promising therapeutic effects of stem cell‐based interventions in conditions such as chronic lung disease, intraventricular hemorrhage, and HIE. These benefits are largely attributed to the cytoprotective, reparative, and regenerative properties of stem cells. Recent evidence indicates that much of their therapeutic effect is mediated indirectly through paracrine mechanisms, particularly involving extracellular vesicles (EVs).^9^

EVs, a heterogeneous group of membrane‐bound particles, have emerged as a novel therapeutic platform with the potential to match the efficacy of whole‐cell therapies while avoiding some of their inherent risks. They play a critical role in intercellular communication by delivering bioactive molecules, including proteins, lipids, and nucleic acids, to target cells, thereby modulating cellular responses and tissue repair processes.^10^ While initial research primarily explored EVs derived from human and animal cells, including stem, progenitor, and stromal populations, more recent studies have identified alternative sources such as plants, fungi, and bacteria, expanding the scope of potential biomedical applications.^11^

The present review aims to provide a comprehensive and critical evaluation of the current evidence regarding the biological actions and molecular mechanisms of EVs derived from diverse sources. It synthesizes data from animal models simulating neonatal diseases, highlights therapeutic implications, and addresses the key challenges that must be overcome to enable successful translation of EV‐based strategies into clinical neonatology.

## 2 BIOGENESIS AND CLASSIFICATION OF EVs

EVs are nanoscale to microscale particles enclosed by a lipid bilayer, actively secreted by virtually all cell types into the extracellular environment. They encapsulate a diverse range of biomolecules, including proteins, nucleic acids, and lipids, and serve as important mediators of intercellular signaling, modulating physiological functions and immune responses.^12^ Previous studies have shown that EV release is a universal process across different cell types, facilitating communication between neighboring and distant cells.^13^

Historically, EV classification was based primarily on their biogenesis pathways, leading to the identification of three principal subtypes: (1) exosomes (40–150 nm), generated via the endosomal system through the fusion of multivesicular bodies with the plasma membrane; (2) microvesicles or ectosomes (100–1000 nm), formed by direct outward budding of the plasma membrane; and (3) apoptotic bodies (100–5000 nm), produced during programmed cell death.^14^

However, advancements in analytical technologies have revealed significant challenges in isolating pure EV subpopulations and accurately determining their intracellular origin.^12^ Additionally, novel vesicle subtypes have been identified, prompting a re‐evaluation of classification criteria. The International Society for Extracellular Vesicles (ISEV) has since proposed the “Minimal Information for Studies of EVs” (MISEV) guidelines, advocating for classification based on physical parameters (size, buoyant density, lipid bilayer presence), biochemical composition (e.g., CD63, CD81, annexin A5 expression), and origin‐related characteristics (e.g., podocyte‐derived EVs, hypoxia‐induced EVs, large oncosomes).^15^ According to the refined nomenclature, small EVs measuring less than 200 nm include subcategories such as exomers (≤50 nm) and supermers (≥25 nm, which lack a lipid bilayer), along with classical exosomes (40–130 nm) and defensosomes (~80 nm). Large EVs, defined as greater than 200 nm, comprise microvesicles (100 nm–1 μm), migrasomes (500–3000 nm), apoptotic bodies (50 nm–5 μm), and large oncosomes (1–10 μm).^16^

Emerging research has emphasized the functional potential of the newly characterized exomers and supermers, which have been less studied compared to classical EV types. Preliminary proteomic analyses indicate that exomers are enriched in proteins linked to hypoxic stress responses, coagulation cascades, and metabolic processes, including glycolysis and mTOR pathway regulation.^17^ In contrast, supermers show significant enrichment in clinically relevant molecules such as amyloid precursor protein (APP), mesenchymal–epithelial transition factor (MET), and glypican‐1 (GPC1), suggesting their role in disease‐related intercellular signaling.^18^ Among all EV classes, exosomes are the most extensively characterized, largely due to their biochemical stability and efficiency in molecular cargo delivery. Their lipid bilayer, composed of phospholipids, cholesterol, and phosphatidylethanolamines, protects the vesicular contents from enzymatic degradation, ensuring structural integrity during transport and enabling long‐distance molecular communication.^19^

## 3 MOLECULAR CARGO OF EVs

EVs are capable of transporting a broad spectrum of bioactive molecules, including proteins, various forms of RNA and DNA, complex carbohydrates, and lipids. In certain contexts, intact organelles or their components, such as mitochondria, have also been detected within EVs. Among their diverse cargo, proteins and microRNAs (miRNAs) are particularly influential in regulating the biological activities of recipient cells.^20^

Extensive proteomic and transcriptomic datasets of EV content are systematically cataloged in specialized repositories such as EVpedia, ExoCarta, and Vesiclepedia, facilitating bioinformatics‐driven functional analyses. For instance, proteomic profiling of mesenchymal stem cell‐derived EVs (MSC‐EVs) from bone marrow has identified over 1900 proteins via mass spectrometry, many of which are involved in cell self‐renewal, differentiation, migration, proliferation, and angiogenesis, particularly under hypoxic conditions.^21^ Similarly, proteomic studies of human placental MSC‐EVs have revealed 745 distinct proteins implicated in angiogenesis, neurogenesis, immunomodulation, and protection against apoptosis and oxidative damage. Notably, many of these proteins participate in key neuroprotective signaling cascades, such as the Wnt and PI3K/Akt pathways.^19^

MSC‐EVs exert neuroprotective effects through two principal mechanisms:

1. *Promotion of neuronal survival and neuroplasticity*: achieved by transferring trophic factors such as nerve growth factor (NGF), brain‐derived neurotrophic factor (BDNF), and glial cell line‐derived neurotrophic factor (GDNF).^22^
2. *Modulation of neuroinflammation*: through the delivery of anti‐inflammatory mediators, including interleukin‐10 (IL‐10), transforming growth factor‐beta (TGF‐*β*), and tumor necrosis factor‐inducible gene 6 protein (TSG‐6).^23^

Additionally, MSC‐EVs have been shown to transfer interleukin‐1 receptor antagonist (IL‐1RA), a critical inhibitor of inflammation‐induced tissue injury. Under pro‐inflammatory stimulation (e.g., IFN‐γ exposure), bone marrow‐derived MSCs produce EVs enriched with neuroprotective extracellular matrix proteins such as laminin *β*2, aggrecan, testican‐1, and periostin, alongside miRNAs with potent anti‐inflammatory activity, including miR‐200b and miR‐146b.^24^

Experimental evidence indicates that the therapeutic potential of MSC‐EVs relies on the synergistic presence of both protein and miRNA components. For example, the degradation of miRNAs reduces the neuroprotective efficacy of MSC‐derived extracellular vesicles (MSC‐EVs) in animal models of autoimmune encephalomyelitis. Several miRNAs with established neuroprotective roles, such as miR‐133b, miR‐21‐5p, miR‐22‐3p, miR‐31, and miR‐146a‐5p, are consistently identified in human MSC‐EVs, highlighting their contribution to tissue repair and functional recovery in neurological disorders.^25^

It is well established that EVs produced by MSCs and a wide variety of other cell types contain an extensive repertoire of RNA molecules. These include messenger RNAs (mRNAs), miRNAs, long non‐coding RNAs (lncRNAs), small interfering RNAs (siRNAs), PIWI‐interacting RNAs (piRNAs), ribosomal RNA fragments (rRNAs), Y‐RNAs, small nuclear RNAs (snRNAs), and circular RNAs (circRNAs). CircRNAs, which belong to the non‐coding RNA family, are generated through a non‐linear backsplicing process involving exons, introns, or combinations of both.^26^ They may enable the translation of small, unique peptides via internal ribosome entry site‐dependent mechanisms. Proper regulation of circRNA expression is critical for maintaining normal neurogenesis, while dysregulation has been associated with the modulation of cellular processes such as autophagy, proliferation, apoptosis, and cell cycle control. Growing evidence links altered circRNA expression to the pathogenesis of neurodegenerative and cardiovascular disorders (Table 1).^25^

**TABLE 1:** Biogenesis, classification, and molecular cargo of EVs.

EV subtype	Biogenesis pathway	Size range (nm/μm)	Key molecular cargo	Functional roles	References
Exosomes	Endosomal pathway; multivesicular bodies fuse with plasma membrane	40–130 nm	Tetraspanins (CD63, CD81), miRNAs (miR‐21, miR‐146a), proteins (TSG‐6, BDNF), lipids (phosphatidylserine, ceramide)	Long‐distance signaling, neuroprotection, anti‐inflammation, angiogenesis	[25]
Microvesicles/ectosomes	Direct outward budding of plasma membrane	100–1000 nm	Integrins, cytokines, growth factors, phosphatidylserine	Cell–cell communication, immune modulation, coagulation	[27]
Apoptotic bodies	Plasma membrane blebbing during apoptosis	100 nm–5 μm	DNA fragments, histones, organelles, apoptotic markers	Clearance of apoptotic debris, immune tolerance	[28]
Exomers	Novel small EV subtype; lipid bilayer‐enclosed	≤50 nm	Hypoxia‐response proteins, glycolytic enzymes	Hypoxic stress response, metabolic regulation	[19]
Supermers	Lipid bilayer–deficient particles	≥25 nm	APP, MET, GPC1	Disease‐related signaling, potential biomarkers	[27]
Migrasomes	Released from retraction fibers during cell migration	500–3000 nm	Signaling proteins, lipids	Intercellular signaling during migration	[29]
Large oncosomes	Shed by aggressive cancer cells	1–10 μm	Oncoproteins, DNA, oncogenic miRNAs	Tumor progression, metastasis	[30]
Specialized EVs (e.g., defensosomes)	Host‐defense vesicles induced by stress/infection	~80 nm	Antimicrobial proteins, immune‐modulatory RNAs	Protection against pathogens	[19]

LncRNAs are transcripts longer than 200 nucleotides that do not encode proteins. They can be classified into several subtypes: sense, antisense, bidirectional, intronic, and intergenic, based on their genomic position relative to protein‐coding genes. LncRNA expression is generally low and highly cell‐type specific. They influence gene expression during development and differentiation, often by regulating chromatin structure and methylation at the post‐transcriptional level, though their precise mechanisms of action remain incompletely understood. Only a small subset of functionally characterized lncRNAs has been identified in EVs, including those found in human breast milk.^31^ Examples include growth arrest–specific 5 (GAS5), steroid receptor RNA activator 1 (SRA1), and colorectal neoplasia differentially expressed (CRNDE). These molecules play important roles in neonatal metabolic regulation. GAS5, for instance, inhibits glucocorticoid‐responsive gene activation during nutrient deprivation or stress, potentially conserving energy in neonatal cells. SRA1 serves as a coactivator of peroxisome proliferator‐activated receptor gamma (PPARγ), a key regulator of adipogenesis, while CRNDE participates in signaling pathways mediated by insulin and insulin‐like growth factor.^32^ Additional lncRNAs in breast milk are implicated in immune regulation. One example, differentiation antagonizing non‐protein coding RNA (DANCR), modulates the expression of interleukin‐6 (IL‐6) and tumor necrosis factor (TNF) genes in monocytes. Furthermore, metastasis‐associated lung adenocarcinoma transcript 1 (MALAT1), present in MSC‐derived EVs, has been linked to the regulation of inflammatory pathways, suggesting therapeutic potential for acute brain injury.^33^

PiRNAs are short RNA sequences of approximately 21–35 nucleotides that function in association with PIWI‐family proteins. These complexes are essential for preserving stem cell genomic stability, primarily through the suppression of retrotransposon activity. Specific piRNAs, such as hsa_piR_017723_DQ594464 and hsa_piR_020814_DQ598650, have been identified in MSC‐derived EVs from human bone marrow, where they protect umbilical cord blood–derived stem cells from apoptotic cell death.^19^

Y‐RNAs represent another class of non‐coding RNAs, less extensively characterized but with notable enrichment in the brain and heart. They are implicated in DNA replication, RNA transcript stabilization, and cellular stress responses. For example, Y‐RNA‐1, which is highly enriched in MSC‐EVs, contributes to hepatocyte protection against TNF‐α/actinomycin D–induced apoptosis. Silencing Y‐RNA‐1 diminishes this protective effect in vitro. Similarly, exosomes from cardiosphere‐derived cells contain Y RNA fragments such as EV‐YF1, which have demonstrated cardioprotective activity following myocardial infarction.^34^

Lipids play a dual role in EV biology, forming the structural architecture of the lipid bilayer and serving as signaling molecules. For instance, cholesterol‐enriched EVs from T‐lymphocytes can stimulate TNF‐*α* production in peripheral blood mononuclear cells, promoting proinflammatory activity. EV lipid cargo includes sphingomyelin, cholesterol, lysophosphatidylcholine, arachidonic acid, fatty acids, prostaglandins, and leukotrienes.^27^ Lipid composition is a critical determinant of vesicle stability, formation, and bioactivity. In exosomes, bis(monoacylglycero)phosphate and ceramides are structural mainstays, while phosphatidylserine translocation to the outer leaflet, together with cholesterol or sphingomyelin presence, is necessary for microvesicle budding.^35^ Lipid classes can also regulate cargo selection; ceramides promote miRNA incorporation, whereas phosphatidic acid facilitates the loading of proteins such as angiopoietin. Recognition and uptake by recipient cells are often mediated by lipid–protein interactions, with phosphatidylserine binding to receptors like Tim1 and Tim4 being a prominent example.^36^

Importantly, EVs from different cell types exhibit distinct lipid profiles, and these differences can serve as diagnostic markers distinguishing healthy from diseased states. Enzymes involved in lipid metabolism are also found within EV proteomes, contributing to the generation of bioactive lipid mediators. For example, dendritic cells and macrophages produce vesicles containing leukotriene C4 synthases, which participate in proinflammatory lipid signaling.^37^ Culture conditions can modify EV lipid content; bone marrow‐derived MSCs exposed to polyunsaturated fatty acids such as arachidonic acid, eicosapentaenoic acid, or docosahexaenoic acid incorporate these lipids into their vesicles, potentially enabling the synthesis of specialized pro‐resolving mediators like resolvin D2. Microglial cell–derived EVs may contain endocannabinoids that modulate neurotransmitter release, thus influencing neuronal excitability.^19^

As research on EV molecular composition advances, a deeper understanding of their role in intercellular communication and disease pathophysiology is emerging. This progress paves the way for novel diagnostic and therapeutic approaches that exploit EV cargo. However, the inherent heterogeneity of vesicle contents, and the context‐dependent responses of recipient cells, introduce complexity into predicting biological outcomes. Functional effects depend not only on the molecules within EVs but also on the physiological state, signaling history, and microenvironment of target cells.^10^

Preserving EV cargo integrity during circulation and storage is a critical challenge, as exposure to enzymes, pH shifts, or environmental stress can degrade active molecules and diminish therapeutic potency. Strategies to enhance stability include modifying vesicle membranes or incorporating protective proteins to safeguard cargo during transit.^27^ Storage methods also influence cargo preservation; for example, freezing EVs in phosphate‐buffered saline may cause ice crystal damage, pH alterations, and ionic imbalances, while lyophilization can produce similar mechanical injury. Current research is exploring cryoprotectants such as Hanks' Balanced Salt Solution with Poloxamer 188 or trehalose as promising alternatives for long‐term vesicle storage.^38^

EVs mediate cell–cell communication by delivering functional molecules to recipient cells through direct fusion, membrane integration, or endocytosis. Uptake mechanisms may involve phagocytosis, macropinocytosis, or receptor‐mediated pathways such as clathrin‐, lipid raft‐, and caveolin‐dependent endocytosis.^39^ Surface molecules like tetraspanins, sialic acids, and major histocompatibility complex proteins confer specificity in target recognition, enabling interactions with neurons, immune cells, or tumor cells. This specificity has been exploited in engineered EVs, such as those modified with rabies virus glycoprotein for targeted delivery to neurons, which in animal models have reduced amyloid burden and inflammation.^40^

## 4 SOURCES OF EVs

The therapeutic potential of EVs depends greatly on their cellular origin. Selecting an appropriate source can enhance targeting specificity and functional outcomes in treating pathological conditions. EVs can be harvested from cultured primary cells, genetically engineered cells, and a variety of human biofluids. Beyond animal‐derived vesicles, plant, bacterial, and fungal EVs are gaining attention as alternative therapeutic platforms, broadening the scope of potential applications.^41^

Conditioned culture medium is one of the most widely utilized reservoirs for obtaining EVs, and several isolation techniques can be applied to recover them. Employing specialized culture formulations has been shown to enhance EV output compared to standard growth conditions. In therapeutic research targeting neonatal disorders, most investigations collect EVs from culture supernatants of stem cells or progenitor cells.^12^ Among all cellular origins, MSCs represent the most thoroughly characterized and frequently exploited source. MSCs can be harvested from bone marrow, adipose tissue, and postpartum tissues such as umbilical cords or placentas. Adipose tissue‐derived MSCs are particularly valuable as an autologous source, being relatively easy to access and yielding substantial vesicle quantities.^42^ These cells have been well characterized biologically, and their therapeutic activity has been validated in preclinical models of neonatal conditions including BPD, HIE, NEC, congenital retinal disorders, and sepsis. Perinatal tissue‐derived MSC‐EVs have also shown notable potential for treating acute brain injuries in newborn models. EVs can originate from either autologous or allogeneic material; however, given that many neonatal pathologies present as medical emergencies, producing autologous MSC‐EVs is often impractical. The cultivation and preparation of autologous vesicle products can take 2–3 weeks, making allogeneic preparations the more feasible option in acute care scenarios.^19^, ^43^

Although MSCs remain the primary focus of EV research, other cellular sources with therapeutic promise have been identified. Immune cell‐derived vesicles, including those from T lymphocytes, B lymphocytes, and macrophages, contribute to immune signaling, pathogenesis, and regulatory processes. For example, macrophage‐derived EVs enriched in CD14 have been shown to mitigate multi‐organ injury in septic models. Endothelial progenitor cell‐derived vesicles have demonstrated protective effects in sepsis through the restoration of vascular barrier function.^44^ Vesicles released by human and murine epicardial cells have been reported to prevent myocardial infarction when locally delivered to injured cardiac tissue in neonatal mice. Additionally, EVs isolated from human plasma have exhibited anti‐apoptotic effects on neonatal rat cardiomyocytes exposed to combined glucose and oxygen deprivation. Cultures derived from induced pluripotent stem cells also represent a promising EV source; neuronal and astrocytic vesicles from such cultures have shown neuroprotective effects in experimental models.^45^ Of particular note, EVs from intestinal neural stem cells have provided gastrointestinal protection against NEC in preterm rat models. Current evidence also suggests that the therapeutic potential of tissue‐specific EVs may be influenced by donor age; for instance, kidney‐derived EVs from newborn rats after acute injury produced stronger reparative responses in murine kidneys compared to adult‐derived vesicles, and a similar age‐dependent decline has been observed in the neuroprotective properties of bone marrow MSC‐EVs.^19^

Biological fluids offer another valuable avenue for EV sourcing. Blood, urine, cerebrospinal fluid, and amniotic fluid all contain vesicles that can be collected for therapeutic use. These fluids provide non‐invasive, readily available sources of bioactive EVs for regenerative applications. Breast milk‐ and amniotic fluid‐derived EVs have been proposed as candidates for neonatal therapies.^46^ Nonetheless, EVs from body fluids are inherently heterogeneous, containing vesicles from multiple cell types, and their therapeutic activity may vary depending on the donor. This variability poses challenges for standardizing clinical products. Furthermore, vesicles derived from biological fluids often reflect the physiological or pathological state of their tissue of origin, making them potentially useful as biomarkers for disease (Table 2).^47^ In the realm of neonatal medicine, the therapeutic potential of EVs, particularly those from non‐animal sources, offers exciting possibilities. Recent advancements point to the therapeutic promise of fungal‐derived EVs, which may address various neonatal conditions due to their bioactive properties. However, it is vital to consider the associated risks, especially regarding allergenicity. Certain fungi can produce allergenic proteins, raising safety concerns when utilizing these EVs in sensitive populations such as neonates. Research has shown that specific fungal strains may trigger hypersensitive reactions in vulnerable infants, highlighting the need for comprehensive evaluation of the immunological profiles of these EVs prior to their clinical use. Additionally, promising results from mouse models have led to ongoing clinical trials examining the effects of EVs derived from grapes, which show potential in preventing oral mucositis resulting from chemo‐radiation in head and neck cancer (ClinicalTrials.gov Identifier: NCT01668849).^48^, ^49^ Furthermore, EVs derived from Aloe and Ginger are being tested for their ability to reduce insulin resistance and chronic inflammation in patients with polycystic ovary syndrome (ClinicalTrials.gov Identifier: NCT03493984).^48^, ^49^ Moreover, researchers are investigating plant‐derived EVs for their potential to enhance immune responses in neonates. Early results indicate a significant increase in specific biomarkers, potentially informing therapeutic strategies tailored to this vulnerable group. By addressing both the risks associated with non‐animal EVs and integrating specific clinical trial details, this discussion promotes a balanced understanding of their therapeutic potential and the translational challenges they face in neonatal medicine. This comprehensive perspective is crucial for guiding future research and clinical applications, ensuring the safety and efficacy of these innovative therapies in neonatal care.

**TABLE 2:** Sources, characteristics, and therapeutic applications of EVs.

Source category	Specific EV origin	Key advantages	Therapeutic applications (preclinical/clinical evidence)	Notable limitations	References
Mesenchymal stem cells (MSCs)	Bone marrow MSCs	Well‐characterized; validated therapeutic effects; stable vesicle production	BPD, HIE, NEC, congenital retinal disorders, sepsis, brain injury	Autologous production takes 2–3 weeks (limits acute use)	[43]
	Adipose‐derived MSCs	Easily accessible; high yield; autologous use possible	Similar to bone marrow MSC‐EVs; high vesicle quantity	May require donor screening for safety	[50]
	Perinatal tissue–derived MSCs (umbilical cord, placenta)	Non‐invasive collection; strong neuroprotective potential	Acute neonatal brain injury, NEC	Allogeneic use may carry immune risks	[43]
Immune cell‐derived EVs	Macrophage‐derived EVs	Enriched in CD14; immune modulation	Multi‐organ protection in sepsis	Source‐dependent variability	[51]
	T/B lymphocyte EVs	Immune regulation	Autoimmune disease modulation	Risk of pro‐inflammatory activity	[27]
Endothelial and cardiac cells	Endothelial progenitor EVs	Vascular barrier restoration	Sepsis‐related vascular injury	Limited large‐scale data	[52]
	Epicardial cell EVs	Cardioprotection	Prevent myocardial infarction	Local delivery needed for efficacy	[45]
iPSC‐derived EVs	Neuronal & astrocytic EVs	Patient‐specific potential; neuroprotection	Neurodegenerative models, NEC	Production complexity, cost	[53]
Tissue‐specific EVs (age‐dependent)	Kidney‐derived neonatal EVs	Enhanced reparative potential	Kidney injury repair	Decline in activity with donor age	[54]
Biological fluid–derived EVs	Plasma, urine, CSF, amniotic fluid	Non‐invasive collection; biomarker potential	Cardioprotection, neuroprotection, NEC	Heterogeneous composition, donor variability	[55]
	Breast milk EVs	Nutritional & immune bioactivity	NEC prevention, immune modulation	Donor‐dependent variability	[56]
Non‐animal EVs	Plant‐derived EVs	Low immunogenicity; GI tract stability	NEC protection, intestinal health	Lower mechanistic characterization	[57]
	Fungal EVs	Immune modulation	Anti‐inflammatory potential	Possible allergenicity	[58]
	Bacterial outer membrane vesicles	Potent delivery system	Immunomodulation, sepsis models	Potential pathogenicity	[27]
Isolation method	Differential ultracentrifugation	High yield, cost‐effective	General EV recovery	Co‐isolation of contaminants	[12]
	Size‐exclusion chromatography	High purity	Precision studies	Low yield, dilution	[59]
	Polymer precipitation	Simple, rapid	Broad vesicle capture	Low selectivity	[12]
	Immunoaffinity capture	High specificity	Targeted EV applications	High cost, low yield	[12]

### 4.1 EVs across biological kingdoms

Evidence from multiple studies indicates that EVs are a deeply conserved mechanism of intercellular communication, enabling horizontal genetic exchange not only between different animal species but also across biological kingdoms such as bacteria, fungi, and plants. This cross‐kingdom signaling highlights the potential evolutionary role of EVs as a universal communication system.^60^, ^61^ Vesicles from non‐animal origins have demonstrated the ability to influence mammalian physiology and may serve as innovative therapeutic tools. For example, plant‐, fungal‐, and bacterial‐derived EVs carry biologically active molecules, including nucleic acids and anti‐inflammatory agents, capable of modulating immune responses and supporting tissue repair in human systems. Their generally low immunogenicity and high compatibility further support their biomedical potential.^57^ Plant EVs have been reported to improve intestinal health in NEC models, fungal vesicles can interact with mammalian immune cells to suppress inflammation, and bacterial outer membrane vesicles (OMVs) can transport virulence factors that contribute to neonatal sepsis.^19^

## 5 ISOLATION METHODS AND CONSIDERATIONS

**FIGURE 1:**
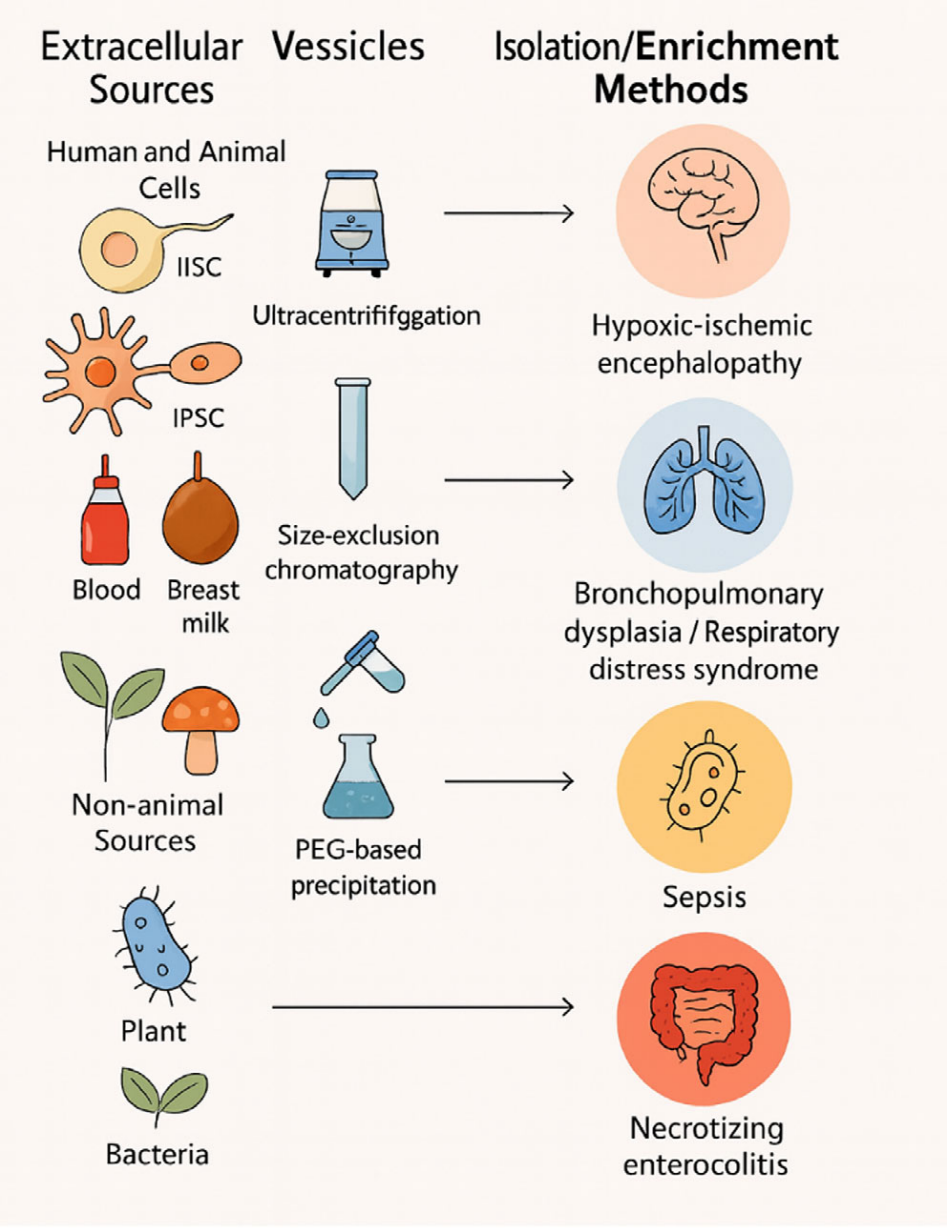
Overview of EV sources, isolation methods, and neonatal disease targets. The left column categorizes EV origins into human/animal cells, body fluids, and non‐animal sources. The middle column illustrates extraction and enrichment techniques with simplified device icons. The right column highlights major neonatal diseases targeted by EV therapies, represented with organ‐specific icons.

In addition to the source, the method of EV isolation is a decisive factor influencing purity, reproducibility, and therapeutic efficacy. Each isolation technique presents its own trade‐offs. Differential ultracentrifugation is a widely used, cost‐effective method that yields high particle numbers but can co‐isolate protein contaminants and other non‐vesicular materials. Size‐exclusion chromatography produces highly purified vesicle populations, though at the expense of yield and with longer processing times that may lead to sample dilution.^12^ Polymer‐based precipitation methods are simple and efficient and can be implemented using commercially available kits, but they lack selectivity, often co‐precipitating vesicles of varying sizes and non‐vesicular particles. Immunoaffinity capture provides unmatched specificity by targeting vesicles with particular surface antigens; however, this method tends to yield smaller quantities and is relatively expensive, and there is a risk of excluding potentially relevant vesicle subtypes. Ultimately, the selection of an isolation approach should be guided by the intended application, balancing factors such as yield, cost, purity requirements, and processing time (Figure 1).^62^, ^63^

## 6 EXPERIMENTAL STUDIES OF EVs AS THERAPEUTIC TOOLS FOR NEONATAL PATHOLOGIES

### 6.1 Hypoxic–ischemic encephalopathy

HIE is a major contributor to neonatal morbidity and mortality worldwide. Epidemiological analyses indicate that HIE affects approximately 2–3 per 1000 live births, accounting for 6%–9% of all neonatal deaths and nearly one‐quarter of mortality in term newborns. Among survivors, about one in four experience severe long‐term neurological complications such as cerebral palsy, epilepsy, seizures, cognitive deficits, and intellectual disability.^64^ The disorder arises primarily from impaired cerebral perfusion and inadequate oxygen delivery to the brain. The injury evolves over time, creating a therapeutic window but also complicating prompt and effective intervention.^65^

HIE pathogenesis is typically described in two stages: primary energy failure and secondary energy failure. The initial stage occurs when reduced cerebral blood flow diminishes oxygen and glucose supply, impairing mitochondrial ATP synthesis and elevating lactate levels. Ion channel dysfunction follows, disrupting calcium homeostasis and causing excessive glutamate release, which triggers further intracellular calcium and sodium influx and leads to necrotic cell death. Apoptosis often ensues days later.^66^ The secondary injury phase begins within 6–48 h post‐insult, driven by oxidative stress, excitotoxic mechanisms, and inflammation. Neonates are especially susceptible to oxidative stress because of high oxygen consumption during extrauterine transition and low endogenous antioxidant reserves. Understanding these mechanisms is essential for designing targeted neuroprotective interventions.^65^

While therapeutic hypothermia has emerged as the standard neuroprotective treatment for eligible infants, its benefits are incomplete. It is limited to infants ≥35 weeks' gestation and does not prevent adverse neurodevelopmental outcomes in all cases. These limitations have fueled the search for novel strategies, with regenerative and cell‐based therapies, particularly MSC approaches, showing promise for both reducing mortality and improving long‐term outcomes.^67^

Early research examined the use of stem/progenitor cells in experimental HIE models, showing that systemic administration of bone marrow‐derived MSCs improved neurological performance, enhanced neuronal and oligodendrocyte generation from neural progenitors, and decreased both neuroinflammation and tissue injury.^68^ Similar protective effects were observed with MSCs derived from umbilical cord blood or placental tissue, which reduced brain injury severity and improved survival in acute injury models. However, engraftment and differentiation of transplanted MSCs within the brain were minimal, suggesting that paracrine signaling, primarily via EVs, is the dominant mechanism underlying their therapeutic effects.^69^

Experimental work with MSC‐EVs has revealed potent immunomodulatory and anti‐inflammatory effects, largely attributed to the regulation of mitogen‐activated protein kinase (MAPK) and nuclear factor kappa B (NF‐κB) pathways.^70^ For instance, in neonatal HIE mouse models, human bone marrow MSC‐EVs administered intraperitoneally reduced brain TNF‐*α* levels while increasing anti‐inflammatory TGF‐*β*. Umbilical cord MSC‐EVs have been shown to modulate Toll‐like receptor 4 (TLR‐4) signaling in microglia, preventing NF‐κB inhibitor degradation and MAPK phosphorylation, thereby suppressing microglial activation and gliosis. Moreover, stereotactic delivery of bone marrow MSC‐EVs into the lateral ventricles selectively inhibited the p38 MAPK/NF‐κB‐p65 axis, reducing transcription of inflammation‐related genes such as IL‐6.^43^

Beyond inflammation control, MSC‐EVs support blood–brain barrier (BBB) integrity, enhance mitophagy, and upregulate neurotrophic factor expression. Surface proteins such as annexin A1 contribute to BBB stabilization. In murine HIE models, MSC‐EVs increased brain levels of BDNF, VEGF, and EGF, correlating with higher neuronal and vascular density and prevention of secondary degeneration. Intranasal delivery of placenta‐derived MSC‐EVs similarly mitigated brain injury progression via PI3K/Akt pathway activation.^19^ The neuroprotective role of mesenchymal stem cell‐derived extracellular vesicles (MSC‐EVs) is closely associated with specific miRNAs. For instance, miR‐146a‐5p derived from umbilical cord MSC‐EVs suppresses pro‐inflammatory microglial activity by modulating the IRAK1/TRAF pathway. Additionally, miR‐181b from adipose‐derived MSC‐EVs enhances endothelial cell proliferation and migration, while miR‐126 promotes neurogenesis and reduces apoptosis in models of hypoxic–ischemic encephalopathy (HIE).^71^

Other EV sources in the developing brain, such as microglia and neural progenitors, also exhibit protective roles. M2 microglia‐derived exosomes containing miR‐124 reduce neuronal apoptosis, infarct size, and functional deficits by targeting USP14. Neural progenitor cell EVs carrying miR‐150‐3p regulate caspase‐2 expression, thereby limiting apoptotic cell death.^72^ Collectively, these findings indicate that EV‐based interventions can influence multiple aspects of brain repair, from neurogenesis and synaptic plasticity to inflammation control, offering therapeutic potential not only during acute injury but also for preventing long‐term neurodegeneration.^73^

### 6.2 RDS and bronchopulmonary dysplasia

RDS and BPD are major causes of neonatal respiratory morbidity, often coexisting in preterm infants. RDS results from insufficient surfactant production, which is critical for maintaining alveolar stability. Surfactant deficiency leads to alveolar collapse, impaired gas exchange, and respiratory failure. Incidence is inversely related to gestational age, reaching nearly universal occurrence in extremely preterm infants born at 24 weeks.^74^

BPD, in contrast, is a chronic lung disorder typically developing in infants who undergo prolonged mechanical ventilation and oxygen therapy for RDS. Its pathogenesis involves lung injury from inflammation, oxidative stress, and mechanical trauma, especially high‐frequency ventilation, disrupting normal lung growth and alveolarization. Approximately 25% of very‐low‐birth‐weight infants develop BPD each year, with a high proportion experiencing moderate‐to‐severe forms and a significant risk of poor long‐term outcomes. Current management largely relies on respiratory support, which maintains lung function but does not promote regeneration or maturation.^75^

MSC‐based therapies have demonstrated regenerative potential in preclinical BPD models, enhancing lung repair and function. One key injury mechanism in ventilated neonates is hyperoxia‐induced oxidative stress, which MSC‐EVs can attenuate through antioxidant actions. Lung tissue injury in BPD is also linked to chronic inflammation, fibrosis, vascular remodeling, and pulmonary hypertension. Studies have shown that MSC‐EVs reduce macrophage infiltration, modulate STAT3 signaling, and shift macrophage phenotypes from pro‐inflammatory M1 to anti‐inflammatory M2 states.^76^

In hyperoxia‐induced BPD mouse models, intravenous MSC‐EV administration alleviated lung inflammation and secondary complications such as fibrosis, vascular remodeling, and pulmonary hypertension. Umbilical cord MSC‐EVs have been shown to restore regulatory T‐cell populations in hyperoxic lungs. Protein factors within EVs, such as TSG‐6, appear critical to their efficacy, with VEGF identified as a key mediator of lung regeneration. The activation of hypoxia‐inducible factor‐1α (HIF‐1α) may also contribute to their protective effects.^43^

Type II alveolar epithelial cells (AEC‐IIs) are pivotal in alveolar maintenance, and their apoptosis contributes to BPD pathology. MSC‐EVs have been shown to prevent AEC‐II death in vitro and in vivo, reducing pro‐apoptotic gene expression (Casp1, Bax) in injured lungs. Bone marrow MSC‐EVs protect epithelial cells via miR‐425‐mediated suppression of PTEN, enhancing PI3K/Akt signaling. Human amniotic epithelial cell‐derived EVs (hAEC‐EVs) also show therapeutic potential, though efficacy varies with donor gestational age; EVs from term infants outperform those from preterm donors in promoting alveolar repair and reducing pulmonary hypertension.^19^, ^77^

Additionally, MSC‐EVs can inhibit transdifferentiation of AEC‐IIs to AEC‐Is by downregulating WNT5a, thereby limiting fibrosis. These multifaceted effects—anti‐inflammatory, antioxidant, anti‐apoptotic, and pro‐regenerative—support the further exploration of EV‐based therapies as a regenerative strategy for both acute and chronic neonatal lung diseases.^78^

### 6.3 Neonatal sepsis

Neonatal sepsis refers to a systemic infection occurring within the first 28 days of life, caused by bacterial, viral, or fungal pathogens. Common infectious agents transmitted from the maternal genital tract during delivery include *Group B Streptococcus*, *Escherichia coli*, *Listeria monocytogenes*, and *Haemophilus influenzae*. Less frequently, coagulase‐negative *Staphylococcus* species and *Streptococcus pneumoniae* are implicated. In addition, neonates may acquire infections in healthcare settings, particularly from antibiotic‐resistant nosocomial strains.^79^

The disease mechanism involves the invasion of microorganisms into the bloodstream, resulting in bacteremia, activation of a systemic inflammatory response, and in severe cases, septic shock. Newborns, especially those born prematurely, are at heightened risk due to their immature immune defenses, limited infection‐localization capacity, and reduced preformed antibody levels. Uncontrolled immune activation can rapidly progress to multi‐organ failure and death, particularly in infections caused by multidrug‐resistant organisms.^80^

Despite advances in antimicrobial therapy, complete infection control in neonates remains challenging. Clinical assessments suggest that in roughly one‐third of newborns and preterm infants, selecting an effective antibiotic is difficult, and treatment may fail to halt disease progression or may exacerbate symptoms. Mesenchymal stem cells (MSCs), due to their systemic regenerative and immune‐modulating actions, have emerged as potential adjunctive treatments for sepsis when combined with standard care.^5^ While specific studies on MSC‐derived extracellular vesicles (MSC‐EVs) in neonatal sepsis are lacking, EVs are increasingly recognized as key mediators in the pathophysiology of sepsis, exhibiting anti‐inflammatory, anti‐apoptotic, and immunomodulatory effects in experimental models.^81^

In cases where pathogens breach the BBB, sepsis may progress to bacterial meningitis, a condition with high mortality and morbidity in newborns.^82^ Experimental evidence in neonatal rats with *Escherichia coli*‐induced meningitis has shown that MSC administration reduces bacterial load and mitigates brain injury. Although bacterial clearance from cerebrospinal fluid was modest, treatment with MSC‐EVs markedly diminished neuronal loss, reactive gliosis, and neuroinflammation, accompanied by significant decreases in IL‐1*β* and TNF‐*α* levels in brain tissue.^83^ Notably, while antibiotics alone eradicated the bacteria, they did not sufficiently reduce inflammation or prevent neural damage. However, the combination of antibiotics with MSC‐EVs not only controlled infection but also attenuated inflammation‐induced brain injury. These findings indicate that MSC‐EVs could offer a novel therapeutic avenue for neonatal meningitis through their potent immunomodulatory and neuroprotective properties.^19^

### 6.4 Necrotizing enterocolitis

NEC is a life‐threatening gastrointestinal disorder in neonates, characterized by inflammation and necrosis of the intestinal wall. It occurs predominantly in premature infants and is associated with multiple risk factors, including immaturity of the gut, formula feeding, intestinal ischemia, and abnormal bacterial colonization.^84^ The disease process involves a disruption in the balance between immune regulation and gut microbiota, triggering an intense inflammatory response that damages intestinal tissue. This can lead to bowel perforation, requiring surgical removal of affected segments and potentially resulting in short bowel syndrome.^85^

Standard management includes cessation of enteral feeding, initiation of total parenteral nutrition, and broad‐spectrum antibiotics. Surgical intervention is necessary in cases with evidence of necrosis or perforation. Despite these measures, outcomes remain suboptimal, and there is an ongoing need for targeted therapies addressing underlying disease mechanisms.^84^, ^85^

MSCs have gained attention as a promising option for NEC treatment due to their potent anti‐inflammatory and tissue‐regenerative effects. Preclinical studies demonstrate that both MSC transplantation and MSC‐conditioned medium containing EVs can preserve intestinal mucosal integrity and reduce disease severity. In vitro studies using intestinal epithelial cell models have shown that these therapeutic effects are largely attributable to exosomes present in the conditioned medium rather than other soluble factors.^43^ Comparative studies in experimental NEC models indicate that EVs derived from various stem cell sources, including amniotic fluid MSCs, bone marrow MSCs, neural stem cells, and intestinal epithelial cells, can reduce NEC incidence and severity, with neural stem cell‐derived EVs showing strong efficacy at lower doses.^53^

The pathogenesis of NEC is strongly linked to an exaggerated inflammatory response in immature gut tissue. MSC‐EVs modulate this immune imbalance by interacting with intestinal macrophages, promoting IL‐10 production, inducing M2 macrophage polarization, and suppressing pro‐inflammatory pathways.^86^ Experimental depletion of intestinal macrophages abolishes the protective effects of MSC‐EVs, underscoring their role in macrophage‐mediated regulation. Furthermore, MSC‐EVs have been shown to restore regulatory T‐cell (Treg) populations, shift T‐helper cell balances toward anti‐inflammatory phenotypes, and reduce Th1/Th17‐mediated inflammation.^87^

The cargo of MSC‐EVs contributes significantly to their therapeutic actions. For example, miR‐378a‐5p can downregulate NLRP3 inflammasome components in macrophages, and TGF‐*β*1 within EVs can suppress neutrophil, dendritic cell, and T‐cell activity while inducing regulatory immune phenotypes. MSC‐EVs also protect epithelial cells from oxidative stress‐induced apoptosis, inhibit pro‐apoptotic caspase expression, and enhance epithelial proliferation. They maintain tight junction integrity and preserve the barrier function of various intestinal epithelial cell types, including enterocytes, stem cells, and goblet cells.^27^

Breast milk‐derived extracellular vesicles (MEVs) represent another natural source of protective factors for neonatal gut health. Secreted during lactation by mammary epithelial cells and possibly resident MSCs, MEVs contribute to immune maturation and intestinal epithelial development. They contain miRNAs, such as members of the let‐7 family and miRNA‐148a that inhibit NF‐κB signaling in dendritic cells, as well as proteins like lactadherin and casein peptides that promote epithelial proliferation, neurodevelopment, and anti‐inflammatory responses. Lactoferrin in MEVs provides additional antimicrobial and immunoregulatory benefits (Table 3).^19^

**TABLE 3:** Experimental studies on EVs as therapeutic tools for neonatal pathologies.

Neonatal pathology	EV source	Key mechanisms of action	Experimental model/delivery route	Main therapeutic outcomes
Hypoxic–ischemic encephalopathy (HIE)	MSC‐EVs (bone marrow, umbilical cord, placenta); Microglia‐derived EVs; Neural progenitor cell EVs	Anti‐inflammatory via MAPK/NF‐κB inhibition; BBB stabilization (Annexin A1); PI3K/Akt activation; Neurotrophic factor upregulation (BDNF, VEGF, EGF); miRNA‐mediated modulation (miR‐146a‐5p, miR‐181b, miR‐126)	Neonatal mouse/rats; Intraperitoneal, intranasal, or stereotactic administration	↓ TNF‐*α*, IL‐6; ↑ TGF‐*β*; Suppressed microglial activation; Preserved BBB; Enhanced neurogenesis and angiogenesis; Reduced apoptosis
Respiratory distress syndrome (RDS)/bronchopulmonary dysplasia (BPD)	MSC‐EVs (bone marrow, umbilical cord, adipose); hAEC‐EVs	Antioxidant and anti‐inflammatory; STAT3 modulation; M1→M2 macrophage shift; VEGF‐mediated lung regeneration; PTEN suppression via miR‐425; WNT5a downregulation	Hyperoxia‐induced BPD mouse models; Intravenous delivery	↓ Macrophage infiltration; ↓ Fibrosis and pulmonary hypertension; Restored Treg cells; Preserved alveolar structure; Reduced AEC‐II apoptosis
Neonatal sepsis/meningitis	MSC‐EVs (bone marrow, umbilical cord)	Anti‐inflammatory (↓ IL‐1*β*, TNF‐*α*); BBB protection; Neuroprotection via reduced reactive gliosis; Combination therapy with antibiotics	*Escherichia coli*‐induced meningitis in neonatal rats; Intravenous + antibiotics	Reduced brain injury and inflammation; Preserved neurons; Controlled infection; Improved survival
Necrotizing enterocolitis (NEC)	MSC‐EVs (amniotic fluid, bone marrow, neural stem cells, intestinal epithelial cells); Breast milk EVs (human/bovine); Bacterial OMVs (*Akkermansia muciniphila*, *Lactiplantibacillus plantarum*)	Immune modulation (↑ IL‐10, Treg; M2 polarization); Inhibition of NLRP3 inflammasome; miR‐378a‐5p and TGF‐*β*1‐mediated suppression of pro‐inflammatory cells; Barrier function restoration; Antimicrobial protein delivery (lactoferrin)	Rat/mouse NEC models; Oral or intraperitoneal delivery	↓ NEC incidence and severity; Preserved mucosal integrity; Reduced oxidative stress and apoptosis; Restored microbiota; Enhanced mucus production

Animal studies have shown that MEVs from both human and bovine milk can ameliorate experimental colitis, reduce pro‐inflammatory cytokine production, restore commensal microbiota balance, and enhance mucus layer production. These findings highlight the potential for integrating MEVs into feeding strategies for preterm infants to prevent or mitigate NEC.^88^ Beyond eukaryotic sources, bacterial OMVs derived from commensal gut bacteria also influence neonatal intestinal health. OMVs from beneficial strains such as *Akkermansia muciniphila* and *Lactiplantibacillus plantarum* have been shown to strengthen epithelial barriers, reduce neutrophil infiltration, and modulate immune responses in colitis models.^89^ Certain OMVs can deliver miRNAs to macrophages, suppressing pro‐inflammatory signaling via MAPK and NF‐κB pathways. These findings suggest that targeting vesicle‐mediated microbiota–host interactions may offer additional avenues for NEC prevention and treatment.^90^

## 7 EMERGING TRENDS IN EVs THERAPY

The field of EV research has rapidly evolved, particularly with the advent of engineering technologies that enhance their therapeutic potential. Engineered EVs offer the ability to modify the natural cargo and surface properties of EVs for targeted therapeutic applications. Recent advancements in genetic and biochemical engineering allow for precise modifications to both the EV membrane and the molecular contents, fostering greater specificity in targeting diseased tissues and cell types. One significant trend is the development of EVs that are engineered for enhanced drug delivery. By incorporating therapeutic agents, ranging from small molecules to RNA‐based therapies, into EVs, researchers can improve bioavailability while minimizing systemic side effects. Targeted EVs can be designed to express specific surface proteins that bind to receptors overexpressed on pathological cells, such as tumor cells or inflamed tissues, thereby increasing the therapeutic efficacy while reducing off‐target effects. For example, EVs derived from mesenchymal stem cells have shown great promise in their ability to deliver gene therapy or siRNA to target cells involved in neonatal diseases. Moreover, the engineering of EVs extends to their release mechanisms. Techniques such as electroporation or the use of stimuli‐responsive release systems can facilitate the controlled release of therapeutic cargo upon reaching specific microenvironments, thus maximizing therapeutic impact at the site of interest. In parallel with engineering advancements, artificial intelligence (AI) is emerging as a transformative technology for analyzing EV cargo. Traditional methods of cargo assessment, such as mass spectrometry and RNA sequencing, often generate vast amounts of complex data. AI algorithms can optimize the processing and interpretation of this data, providing insights that are otherwise difficult to achieve through manual analyses. AI‐driven approaches include the application of machine learning models to predict the functional outcomes of specific EV cargo based on large datasets. These models can identify key biomolecules associated with particular therapeutic effects, aiding in the design of EVs tailored for specific clinical applications. For instance, by analyzing the proteomic and genomic profiles of EVs, AI can assist in determining which combinations of proteins and RNAs are most effective in modulating immune responses or promoting tissue regeneration in neonates. Furthermore, integrating AI with imaging techniques allows for the real‐time tracking of engineered EVs within biological systems. This capability enhances our understanding of EV biodistribution, uptake, and therapeutic efficacy in neonatal models, enabling a dynamic and holistic view of how modified EVs interact with target tissues. The incorporation of engineering strategies in the development of EVs, paired with AI‐driven data analysis techniques, represents a significant shift in therapeutic approaches. These emerging trends not only enhance the understanding of EV biology but also pave the way for innovative treatments that could substantially improve outcomes in neonatal medicine. As we continue to explore these advancements, it becomes increasingly clear that engineered EVs and AI methodologies could serve as pivotal tools in addressing the complex challenges present in neonatal healthcare.

## 8 CHALLENGES OF TRANSLATING EVs INTO CLINICAL PRACTICE

Experimental findings from models of neonatal and preterm infant disorders offer compelling evidence supporting the therapeutic potential of EVs and provide a strong platform for advancing translational research toward clinical applications. However, several critical issues must be addressed before EV‐based interventions can be implemented as routine therapeutic options.^27^

A primary consideration is the choice of an optimal EV source with consistently high therapeutic efficacy. Historically, MSCs have been the predominant source investigated in regenerative medicine; however, recent investigations have expanded the repertoire to include alternative origins, such as milk‐derived vesicles and non‐animal EVs, which have shown promising bioactivity.^9^

Another major obstacle is the incomplete characterization of EV biology. While their presence, clinical potential, and some mechanistic pathways have been described, the precise identity and molecular composition of the active therapeutic constituents remain insufficiently defined. The contribution of specific non‐coding RNAs, including piRNAs, ribosomal RNAs, small nuclear and nucleolar RNAs, and circular RNAs, has not yet been fully elucidated.^12^ Similarly, the functional roles of double‐stranded DNA detected within EVs are still unclear. These uncertainties hinder the optimization of EV‐based treatments. To overcome this, in‐depth molecular profiling through advanced proteomic and genomic methodologies is required to accurately identify and quantify the bioactive “cargo” responsible for therapeutic effects.^27^

**FIGURE 2:**
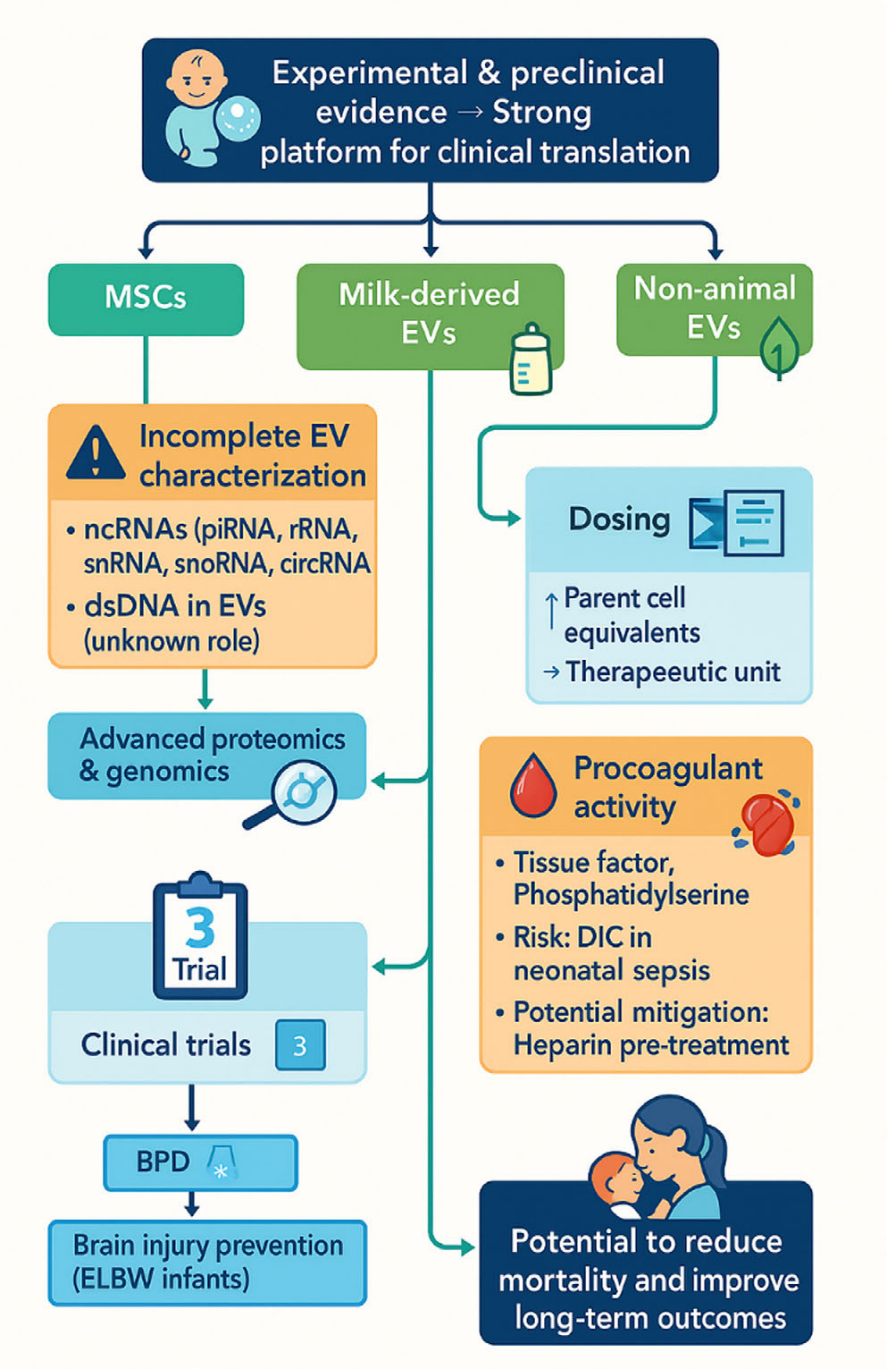
Pathway from EV research to clinical impact in neonatal therapies.

Standardizing methods for EV isolation, purification, and characterization is also essential to ensure reproducibility and cross‐study comparability. Variability in vesicle heterogeneity remains a major challenge, necessitating robust, universally accepted protocols for EV preparation and for qualitative and quantitative evaluation of their components. Equally important is establishing dosing guidelines.^15^ EV quantification currently relies on varied approaches, ranging from parent cell equivalents and total protein content to particle concentration and size analysis, yet no consensus exists on optimal dose, administration frequency, or delivery route for neonatal therapies. Experimental studies to date have often lacked uniform dosing standards, with therapeutic amounts differing by up to two orders of magnitude. The proposal of a “therapeutic unit” as a standardized dosing metric is one attempt to address this, enabling meaningful cross‐study comparisons, but further validation is needed (Figure 2).^91^

Safety considerations are equally critical. EVs can present procoagulant activity due to surface expression of tissue factor and phosphatidylserine, which may activate coagulation cascades. Such properties raise concerns in vulnerable patient populations, particularly neonates with sepsis‐related disseminated intravascular coagulation, where thrombosis and inflammation can be life‐threatening. Although preliminary work suggests that pre‐treatment with agents such as heparin could mitigate these risks, comprehensive preclinical evaluations of EV safety in the context of thrombotic complications are required.^27^ Furthermore, interactions with other therapies must be carefully studied; for example, preconditioning with hypothermia in neonatal HIE models has been reported to diminish subsequent cell‐based therapeutic efficacy.^92^

Despite these complexities, clinical translation is progressing. At present, three registered clinical trials are investigating EV‐based interventions in neonates, targeting conditions such as BPD, prevention of brain injury in extremely low birthweight infants, and treatment of HIE. If these efforts succeed, the incorporation of EV therapeutics into neonatal medicine could markedly reduce mortality and improve long‐term functional outcomes in this highly vulnerable population.^9^

## 9 CONCLUSIONS

EVs hold significant promise as novel therapeutic agents for a range of neonatal pathologies, with MSC‐derived vesicles currently being the most extensively studied and showing the greatest potential.^19^ Nonetheless, gaps remain in the precise molecular characterization of EV cargo and in the full delineation of their mechanisms of action. Addressing these gaps will require rigorous, GLP‐compliant omics‐based profiling to define vesicle composition and function.^93^ Additionally, adherence to standardized guidelines such as the MISEV criteria is necessary to ensure the reproducibility and safety of EV preparations. Careful and well‐controlled preclinical studies, emphasizing safety, dose optimization, and mechanistic clarity, will be pivotal in facilitating a smooth transition from laboratory research to early‐phase clinical trials, ultimately enabling the integration of EV‐based therapies into mainstream neonatal care.^94^

## AUTHOR CONTRIBUTIONS

*Conceptualization and Supervision, Investigation; Data curation; Writing—original draft*: Ali M Atoom, Media Hamed‐Ahmed, Shaker Al‐Hasnaawei, H Malathi, Laxmidhar Maharana, Anima Nanda, Vimal Arora, Ashish Singh‐Chauhan, Elham Poursoltani.

## CONFLICT OF INTEREST STATEMENT

The authors declare that they have no known competing financial interests or personal relationships that could have appeared to influence the work reported in this paper.

## Data Availability

Data sharing not applicable to this article as no datasets were generated or analysed during the current study.
